# Metagenome-Assembled Genomes of *Komagataeibacter* from Kombucha Exposed to Mars-Like Conditions Reveal the Secrets in Tolerating Extraterrestrial Stresses

**DOI:** 10.4014/jmb.2204.04009

**Published:** 2022-07-07

**Authors:** Imchang Lee, Olga Podolich, Bertram Brenig, Sandeep Tiwari, Vasco Azevedo, Daniel Santana de Carvalho, Ana Paula Trovatti Uetanabaro, Aristóteles Góes-Neto, Khalid J. Alzahrani, Oleg Reva, Natalia Kozyrovska, Jean-Pierre de Vera, Debmalya Barh, Bong-Soo Kim

**Affiliations:** 1 Department of Life Science, Multidisciplinary Genome Institute, Hallym University, Chuncheon 24252, Republic of Korea; 2 Institute for Liver and Digestive Diseases, Hallym University, Chuncheon, Republic of Korea; 3 Institute of Molecular Biology and Genetics of NASU, Kyiv 03143, Ukraine; 4 Institute of Veterinary Medicine, Burckhardtweg, University of Göttingen, Göttingen 37073, Germany; 5 Departamento de Genética, Ecologia e Evolução, Instituto de Ciências Biológicas, Universidade Federal de Minas Gerais, Belo Horizonte, Minas Gerais 6627, Brazil; 6 Molecular and Computational Biology of Fungi Laboratory, Department of Microbiology, Institute of Biological Sciences, Federal University of Minas Gerais, Belo Horizonte 6627, Brazil; 7 Department of Clinical Laboratories Sciences, College of Applied Medical Sciences, Taif University, Taif 21944, Saudi Arabia; 8 Centre for Bioinformatics and Computational Biology, Department of Biochemistry, Genetics and Microbiology, University of Pretoria, Pretoria 0028, South Africa; 9 Microgravity User Support Center (MUSC), German Aerospace Center (DLR), Cologne 51147, Germany; 10 Institute of Integrative Omics and Applied Biotechnology (IIOAB), Nonakuri, Purba Medinipur, WB, 721172, India; 11 The Korean Institute of Nutrition, Hallym University, Chuncheon 24252, Republic of Korea

**Keywords:** Kombucha, *Komagataeibacter*, metagenome assembled genome, mars-like condition, whole metagenome

## Abstract

Kombucha mutualistic community (KMC) is composed by acetic acid bacteria and yeasts, producing fermented tea with health benefits. As part of the BIOlogy and Mars EXperiment (BIOMEX) project, the effect of Mars-like conditions on the KMC was analyzed. Here, we analyzed metagenome-assembled genomes (MAGs) of the *Komagataeibacter*, which is a predominant genus in KMC, to understand their roles in the KMC after exposure to Mars-like conditions (outside the International Space Station) based on functional genetic elements. We constructed three MAGs: *K. hansenii*, *K. rhaeticus*, and *K. oboediens*. Our results showed that (i) *K. oboediens* MAG functionally more complex than *K. hansenii*, (ii) *K. hansenii* is a keystone in KMCs with specific functional features to tolerate extreme stress, and (iii) genes related to the PPDK, betaine biosynthesis, polyamines biosynthesis, sulfate-sulfur assimilation pathway as well as type II toxin-antitoxin (TA) system, quorum sensing (QS) system, and cellulose production could play important roles in the resilience of KMC after exposure to Mars-like stress. Our findings show the potential mechanisms through which *Komagataeibacter* tolerates the extraterrestrial stress and will help to understand minimal microbial composition of KMC for space travelers.

## Introduction

Kombucha is one of the most popular healthy beneficial beverage consumed in all over the world [1, 2]. The effects of Kombucha and its ingredients on health has been reported in several studies [1-3]. Recently, the microbiome of Kombucha mutualistic community (KMC) has been largely studied to understand the microbial roles in the beneficial effects of Kombucha. Although the microbial composition is varied among different KMCs, dominant members (*Brettanomyces*, *Pichia*, *Saccharomyces*, *Schizosaccharomyces* and *Zygosaccharomyces* as yeast members, and *Acetobacter*, *Lactobacillus*, *Leuconostoc*, *Bifidobacterium*, and *Komagataeibacter* as bacterial members) were the most common representatives [2-5]. *Komagataeibacter* species have been focused on as dominant bacteria that produce cellulose, gluconic, and glucuronic acid by fermentation in KMCs [6, 7]. The microbiome, which consists of various microbes, is a dynamic ecosystem that shares genetic elements with each other and responses to their habitat’s changes. Among the members, substantially essential organisms for controlling the function and maintenance of the microbiome are called keystone taxa and dominant taxa [8]. Therefore, analysis of the keystone taxa is important to understand the shift of the microbiome and to determine the minimal composition of the microbes in a stressed environment.

In the BIOMEX (Biology and Mars Experiment) project, Kombucha samples were used as a material to understand the influence of exposure to spaceflight and Mars-like conditions on microbiome of Kombucha. The composition of microbiome in KMCs and the effect of long-term exposure to a harsh stressful environment of Martian conditions on dried and partially mineralized cellulose-based KMC biofilms in low Earth orbit were analyzed in our previous studies [9, 10]. In the studies, *Komagataeibacter* was determined as the dominant genus in KMCs exposed to Mars-like conditions, and the cellulose-based biofilm produced by genus *Komagataeibacter* was key material to protect cells from germicidal environments like UV and dried state which were generated Mars-like conditions. Although the microbiome and the importance of *Komagataeibacter* in KMCs exposed to harsh conditions were reported, genetic characteristics of *Komagataeibacter* in KMCs according to exposure conditions by using metagenome-assembled genome (MAG) analysis is not yet done to understand the genomic functionality in extra-terrestrial conditions.

In this study, we reconstructed and analyzed the high-quality MAGs in KMCs exposed to Mars-like conditions outside the International Space Station (ISS) to understand the role of *Komagataeibacter* through the lens of its MAG in survival and to define how the *Komagataeibacter* retains its dominance in KMC under stressful Martian conditions.

## Materials and Methods

### Samples, Space Exposure Conditions, and Metagenome Sequencing

**Fig. 1:**
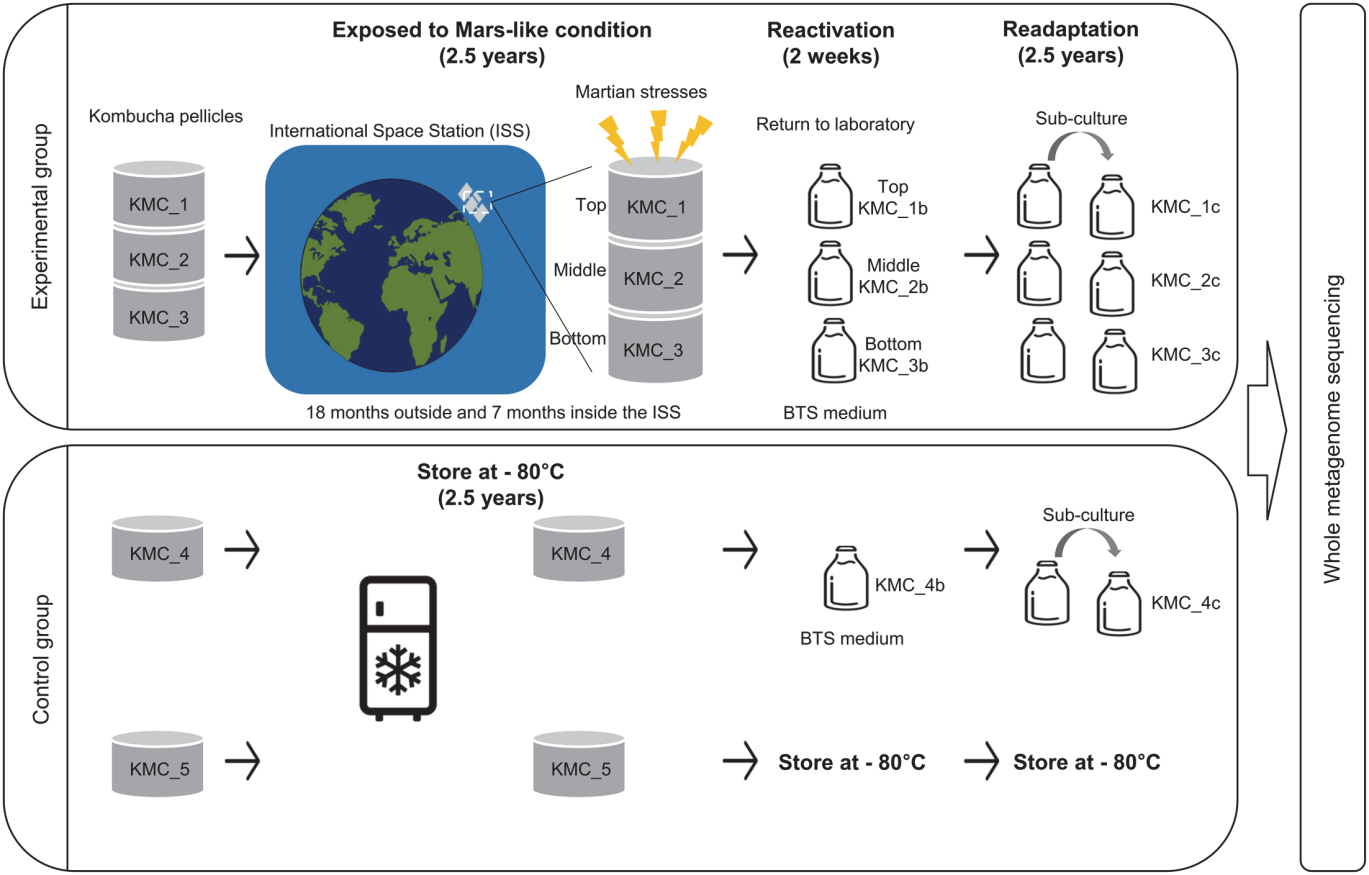
Summary of experimental schemes in this study.

Experimental conditions for exposure, reactivation, and readaptation of KMC samples and laboratory references were described in our previous study [10]. Briefly, dried KMC pellicles from collection of Institute of Molecular Biology and Genetics (IMBG, Ukraine) were exposed to Mars-like conditions in the three-layer sample carrier on the EXPOSE-R2 facility outside the ISS (Fig. 1). Samples were exposed to UV (> 200nm), cosmic ionizing radiation, and other fluencies during 2.5-year (18 months outside and 7 months inside the station). Returned KMC pellicles from the ISS were cultured in black tea with white sugar (BTS) medium during 2 weeks for reactivation, and reactivated samples were sub-cultured in BTS medium during 2.5-year for readaptation. Analyzed KMC samples consist of nine samples including one initial KMC sample (KMC_5), three post-flight reactivated samples (KMC_1b, KMC_2b, and KMC_3b) exposed on top, middle, and bottom levels of the carrier, and three readapted samples (KMC_1c, KMC_2c, and KMC_3c). The top-layer sample was exposed to UV and the samples of middle and bottom layer were maintained unlighted and UV-protected condition during the exposure period. In parallel, laboratory-kept controls, KMC_4b (reactivated along with the returned from the ISS) and KMC_4c (cultured for 2.5-year with KMC_4b) were used as ground-based references. After reactivation, the aliquots of post-flight samples and control samples (laboratory kept sample: KMC_4b and the initial KMC ecotype: KMC_5) were stored at -80°C. The metagenomic DNA extraction and whole metagenome sequencing for all the samples were performed as described in our previous study [9]. The shotgun metagenome sequences were obtained from Illumina HiSeq 2500 (150-bp paired ends).

### Analysis of Functional Gene Profiles in Whole Metagenome Sequences Based on Short-Reads

Functional gene profiles in KMC samples were analyzed by using the HMP Unified Metabolic Analysis Network (HUMAnN 3.0) tool [11]. The differences of profiles between the samples were analyzed in the nonmetric multidimensional scaling (NMDS) plot [12] based on the Bray-Curtis dissimilarity matrix [13]. Fifty iterations were performed and the resulting ordination that had the lowest stress was used for data visualization. The monoMDS function implemented by the vegan package of R was used for MDS [14]. Taxonomic identification for functional genes and the conversion of functional gene features to KEGG Orthology (KO) were obtained from the HUMAnN. The relative abundance of species was normalized with copies per million (CPM) and unmapped reads were excluded for comparison among samples. The changed KOs in each KMC sample compared to initial KMC sample (KMC_5) were analyzed by heatmap plot.

### Reconstruction of the MAGs

The co-assembly of the quality-filtered reads was performed using MEGAHIT ver. 1.2.9 [15], and the contigs shorter than 1 kb were discarded. After the co-assembly process, the rest of the sequencing reads were mapped to the assembled contigs using Bowtie2 ver. 2.4.1 [16]. The co-assembled and mapped reads were binned based on the single-copy core gene (SCG) set of Kaiju ver. 1.6.2 [17] using Anvi’o ver. 6.2 [18]. The Anvi-refine inherent in the Anvi’o was used to identify pairs of bins. The completeness and contamination of the reconstructed bins were estimated using CheckM ver. 1.1.3 [19]. MAGs with an estimated completeness > 90% and contamination < 5%were used for further analyses.

### Classification of MAGs

The reconstructed MAGs were initially classified with SCG of Kaiju program and the detected SCG were identified using BLASTP program [20] with the National Center for Biotechnology Information (NCBI)-nr database [21]. For accurate classification of MAGs, the genomic distances between the completed genome sequences of closest strains predicted based on BLASTP results and obtained MAGs were calculated using OrthoANI tool [22]. The genomic nucleotide variation-based phylogenetic trees were used for inferring the evolutionary relationship between MAGs and a given reference type strains using PhaME tool [23]. Bootstrap analysis was performed by resampling 100 times the entire set, and the bootstrap values were displayed on the maximum likelihood phylogeny tree. In order to reduce bias during the reconstruction of the MAGs, only accurately classified MAGs among the reconstructed bins were used for further analysis.

### Assignment of Functional Genetic Elements in MAGs

In order to identify functional genes in MAGs, gene contents in each MAG were assigned at module-level using the GhostKoala ver. 2.2 with default options in the KEGG database ver. 96.0 [24]. The information of the functional operon in the MAGs was obtained from the annotated data generated by using the Prokka program [25]. The sequence data of the reconstructed *Komagataeibacter* MAGs in this study were deposited in the NCBI GenBank database under the accession numbers of GCA_016785065.1, GCA_016785115.1, and GCA_016785145.1.

## Results

### Comparison of Functional Gene Profiles Based on Short-Reads among KMCs Samples

**Fig. 2:**
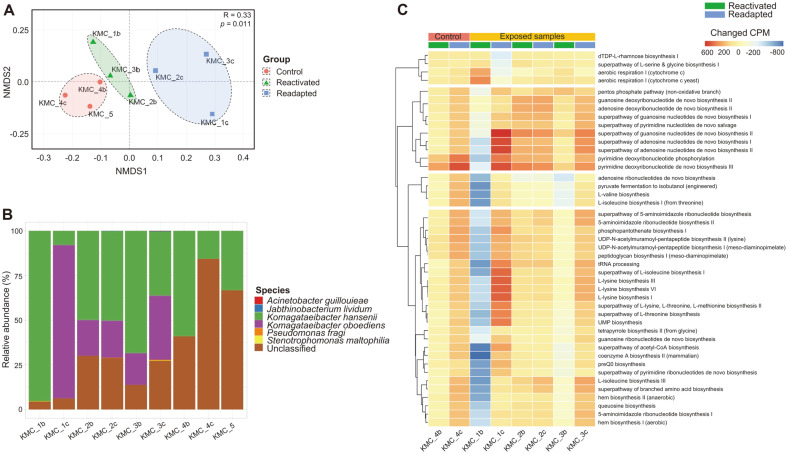
Comparison of functional profiles among KMC samples. (**A**) The difference of functional features in microbiome of KMCs was analyzed by NMDS plot based on Bray-Curtis dissimilarity. Red indicates control group, green indicates post-flight group, and blue indicates readapted post-flight group. (**B**) The relative abundances of species identified by functional genes were compared among KMC samples. The relative abundance of the taxa was normalized using copies per million (CPM), and unmapped reads were excluded in analysis. (**C**) The changed KEEG Ortholog (KO) in KMC samples compared to initial reference sample (KMC_5) were analyzed by heatmap.

The differences of functional gene profiles among KMC samples were analyzed by means of an analysis of similarities (ANOSIM) based on the rank-order Bray-Curtis distance (Fig. 2A). Functional genes of microbiome in samples of a control group (KMC_4b, KMC_4c, KMC_5), a post-flight reactivated group (KMC_1b, KMC_2b, KMC_3b), and a readapted group (KMC_1c, KMC_2c, KMC_3c) were significantly different (*p* = 0.011). Two species of *K. hansenii* and *K. oboediens* were found to be dominant members in all samples, and the relative abundances of these two species were higher in the top-layer samples (95.22% of *K. hansenii* in KMC_1b and 91.89% of *K. oboediens* in KMC_1c) than other samples (Fig. 2B). Functional genes of unclassified taxa were relative higher in laboratory-kept samples (mean value of 63.95%) than KMC samples after exposure Mars-like condition. *K. hansenii* was dominant in laboratory-kept samples (KMC_4b, KMC_4c, and KMC_5) and top-layer of post-flight reactivated sample (KMC_1b). The relative abundances of *K. oboediens* were increased in middle-and bottom-layers of reactivated samples (KMC_2b and KMC_3b) and all layers of readapted samples (KMC_1c, KMC_2c, and KMC_3c). The changed KOs compared to the initial sample (KMC_5) were different between top-layer samples (KMC_1) and other samples (Fig. 2C). The relative abundances of KOs were decreased more in reactivated top-layer sample (KMC_1b) than other samples, whereas their abundances were increased more in readapted top-layer sample (KMC_1c). Most of them were related to biosynthesis of nucleotides and amino acids.

### Reconstruction and Identification of the MAGs: *K. hansenii*, *K. rhaeticus*, and *K. oboediens*

**Fig. 3:**
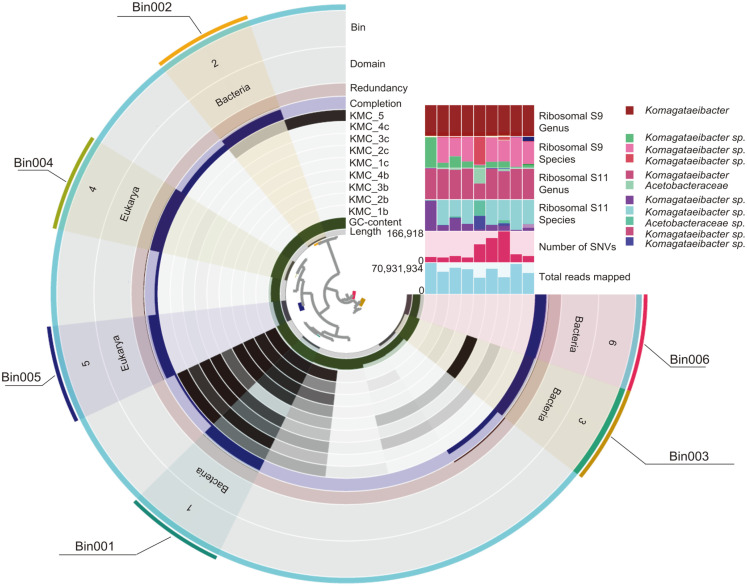
Comparison of bin results from metagenome sequences using Anvi’o program. Dendrogram shows the distribution and taxonomic identification of each bin based on single-copy core gene (SCG) of Kaiju. The first inner circle showed the length of each SCG, and second circle indicated GC contents (%). The following nine circle layers represented the proportion of contigs in each KMC sample. The next layers showed the completeness, redundancy, and taxonomic information of each bin. The proportion of total mapped reads and the number of single nucleotide variation (SNV) in each KMC samples were compared by 5^th^ and 6^th^ of upper-right bar chart. The relative abundance of taxonomic composition by each marker gene set was compared among KMC samples by 1^st^ to 4^th^ of upper-right bar charts.

A total of 497,559,912 reads were co-assembled resulting in 7,303 metagenomic contigs with a mean length of > 65,241 kb. The contigs with short length (< 1,000 bp) were removed from 7,303 contigs, and the resultant 1,435 contigs were used for the binning process. Six bins were retrieved after binning, and 2 bins (bin004 and 005) were removed by quality check of completeness (> 90%) and contamination (< 5%) (Fig. 3). These two bins that were removed, bin004 (89.16% completeness) and bin005 (83.13% completeness), were predicted as *Pichia* (a yeast genus) based on the SCG set of Kaiju. Although bin006 was predicted as *Pseudomonas* with high quality (100%completeness and 1.41% contamination), it was not accurately classified in a detailed analysis to delineate species using taxonomical indices including average nucleotide identity (ANI) value and 16S rRNA gene similarity. Thus, we had excluded bin006 from analysis to reduce misunderstanding by potential biases during MAG reconstruction [26]. As a result, three bins (bin001, bin002, and bin003) were retrieved from all the KMC samples and were predicted as *Komagataeibacter* (bin001 and 003) and Family *Acetobacteraceae* (bin002) by the SCG set of Kaiju. The bin001, bin002, and bin003 were named as kmcMAG001, kmcMAG002, and kmcMAG003 for further analyses.

**Fig. 4:**
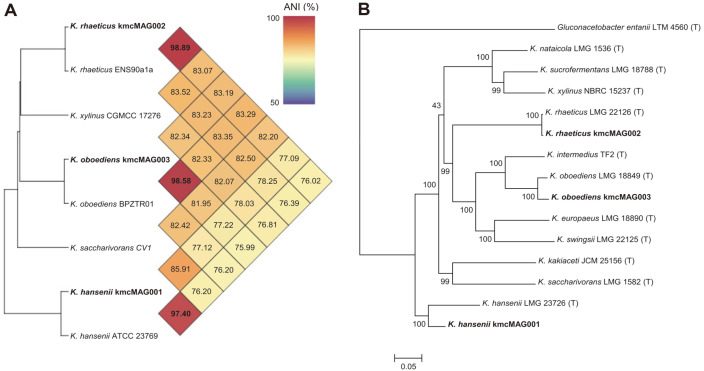
Phylogenetic analysis between MAGs and related taxa. (**A**) The average nucleotide identity (ANI) values were calculated between reconstructed MAGs and the genomes of the closest strains selected by SCGs of Kaiju. (**B**) The Maximum- Likelihood phylogenetic tree of MAGs and genome sequences of the closest species (type strain). The phylogenetic tree was constructed using the PhaMe tool. Bootstrap analysis was performed by resampling 100 times the entire set, and the bootstrap values are displayed on the phylogeny tree.

For accurate classification of these three bins, a phylogenomic analysis was performed with the closest strains. The similarity between MAGs and the genome of the closest strains was calculated by ANI value (Fig. 4A). The ANI value between kmcMAG001 and *K. hansenii* ATCC23769 was 97.40%, and that between kmcMAG002 and *K. rhaeticus* ENS90a1a was 98.89%, and that between kmcMAG003 and *K. oboediens* BPZTR01 was 98.58%. The classification of the MAGs at species-level was determined by genome single nucleotide polymorphism (SNP) tree with the MAGs and the genomes of type strains of the predicted species. As a result, the MAGs were classified as *K. hansenii* (kmcMAG001), *K. rhaeticus* (kmcMAG002), and *K. oboediens* (kmcMAG003) (Fig. 4B). General features of three obtained MAGs were summarized in our previous report [27]. Briefly, genome sizes of the three *Komagataeibacter* MAGs were in a range from 3,326,596 to 4,214,122 bp, and their correspondent GC contents were in a range from 60.14% to 63.35%. The N_50_ values of the MAGs displayed a range of 62,857 to 88,723 bp. Estimated completeness of the kmcMAG001, kmcMAG002, and kmcMAG003 were 94.65%, 99.11%, and 98.77%, respectively, and redundancy (contamination rate) were 4.98%, 1.69%, and 1.49%, respectively.

### Comparison of Functional Gene Features among Three *Komagataeibacter* MAGs

**Fig. 5:**
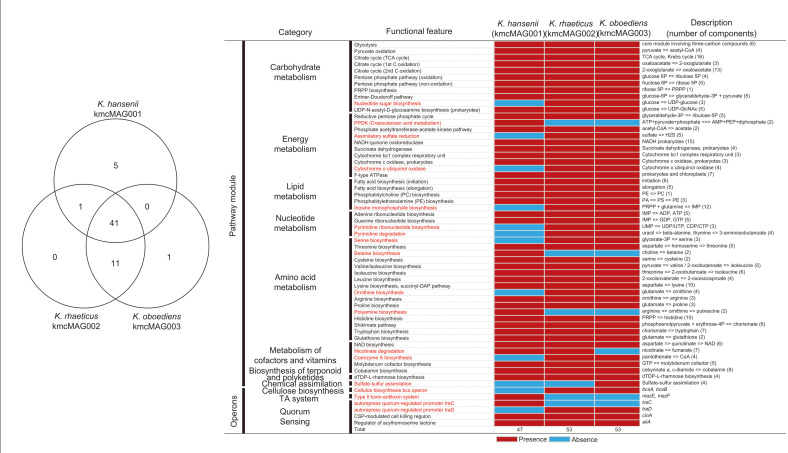
Comparison of the genomes among three *Komagataeibacter* MAGs. A total of 59 functional elements, including 53 pathways and 6 operons, were detected. Venn diagram shows the number of sharing genetic elements among MAGs. The annotation was conducted in module-based pathway and prediction of the operons using GhostKoala in the KEGG database and Prokka, respectively. Red bar indicates the presence of gene contents and blue bar indicates the absence of gene contents in each MAG. The elements varying in presence or absence are highlighted in red text.

The three reconstructed *Komagataeibacter* MAGs were dominant members in all the KMC samples, and they could play as keystone in KMCs. Thus, we compared the gene contents among the three *Komagataeibacter* MAGs (Fig. 5). A total of 59 genetic elements, including 53 completed module-level pathways and six operons were identified in our three MAGs. Forty-one elements were commonly detected in all the MAGs. Five elements, bacterial pyruvate phosphate dikinase (PPDK) related pathway, betaine biosynthesis, polyamine biosynthesis, type II toxin-antitoxin system, and auto-repress quorum-regulated promoter TraC were unique in the *K. hansenii* MAG (kmcMAG001). Functional genetic elements of the *K. rhaeticus* (kmcMAG002) were similar to those in *K. oboediens* (kmcMAG003). One operon (*bcs*: biosynthesis of the bacterial cellulose) and ten genetic elements (nucleotide sugar biosynthesis, cytochrome o ubiquinol oxidase, inosine monophosphate biosynthesis, pyrimidine ribonucleotide biosynthesis, pyrimidine degradation, serine biosynthesis, ornithine biosynthesis coenzyme A biosynthesis, auto-repress quorum-regulated promoter TraD) were commonly detected in *K. rhaeticus* and *K. oboediens* MAGs. The sulfate-sulfur assimilation pathway was unique in the *K. oboediens* (kmcMAG003). Several important operons for the communication between microorganisms were detected in these *Komagataeibacter* MAGs. The competence-simulating peptide (CPS)-modulated cell killing regulon (*cinA*) and regulator of acylhomoserine lactone (*aiiA*), which are components of quorum sensing (QS) related operons, were detected in the three *Komagataeibacter* MAGs. Autorepressive quorum-regulated promoter of the *tra* gene was commonly detected in three MAGs. However, the *traC* was detected only in the kmcMAG001, and the *traD* was in the kmcMAG002 and kmcMAG003. The toxin-antitoxin (TA) system, represented with the type II TA *mazEF* operon, was unique in kmcMAG001.

## Discussion

Although the diversity of KMCs showed fluctuation throughout the samples, the genus *Komagataeibacter* were still regarded as dominant organism of the KMC samples. As the genus *Komagataeibacter* was revealed as a key microorganism in our previous study [9], the analysis to investigate of genetic insight of *Komagataeibacter* is crucial for revealing the core function of KMC microbiomes in stressful environment. Therefore, we analyzed the alteration of functional gene features and reconstructed MAGs from whole metagenome sequences in KMC samples exposed to Mars-like conditions. We compared genetic elements in *Komagataeibacter* MAGs, which was dominant members in all the KMC samples, to understand the tolerance and reactivation of *Komagataeibacter* after exposure to Martian environments. Our results showed that genetic elements in *Komagataeibacter* species had the potential to survive and modulate the other microorganisms in KMC.

The profiles of functional genes in KMC samples were shifted after exposure to Mars-like conditions consist of 95.55% CO_2_, 2.7% N_2_, 1.6% Ar, 0.15% O_2_, ~370 ppm H_2_O and a pressure of 980 Pa [9, 10]. Functional genes in the microbiome of readapted samples (KMC_c) were more different to those of control samples (KMC_4 and KMC_5) than post-flight reactivated samples (KMC_b). These differences of functional features were caused by the alteration of microbiota according to conditions after exposure to space environment. The exposure to Mars-like condition affected the microbiome in KMCs and re-cultured microbiota after exposure was different to the microbiota in non-exposed controls (laboratory-kept samples). The shifted microbiota in KMCs and functional genes were also different according to exposed layer in carrier outside the ISS. The functional genes in the microbiome of top-layer samples (KMC_1) were more different to those of control samples than middle and bottom-layer samples (KMC_2 and KMC_3) in both reactivated and readapted samples. The top-layer sample was exposed to UV radiation which could emit about 4.92 × 102 kJ / m2 and 0.5 Gray (Gy) of cosmic-ionizing radiation, and the samples of middle and bottom layer were maintained unilluminated and UV-protected condition. Therefore, the microbiome in top-layer samples was exposed to strong stressors and their influences were higher than those in other-layer samples. *K. hansenii* was still dominant in reactivated (cultured post-flight samples for 2 weeks) samples. In particular, the proportion of this species was the highest in reactivated sample of top-layer exposure (KMC_1b). *K. oboediens* was dominated in readapted samples (sub-cultured for 2.5-year). The microbiota in middle-layer samples were similar between reactivated and readapted conditions. The proportion of unclassified taxa was decreased in KMCs after exposure to harsh conditions. This indicates that *K. hansenii* was tolerant to harsh condition including UV exposure and could reactivate immediately after return in reactivated samples. However, *K. oboediens* was gradually dominated in repeated cultures for relatively long period (2.5-year). Although the microbiome in KMC was influenced by exposure to Mars-like condition, *Komagataeibacter* spp. survived and dominated in reactivated and readapted samples. In addition, three *Komagataeibacter* MAGs were obtained from whole metagenome sequences with high quality (completeness > 90% and contamination < 5%). We analyzed and compared genomic elements among reconstructed *Komagataeibacter* MAGs in KMC samples.

Two *Komagataeibacter* species were dominant in short-read based result, whereas three *Komagataeibacter* species were obtained in MAGs based result. This difference could be due to the analysis method including assembly and reference databases. In addition, genomic contents in *K. rhaeticus* MAG were similar to those in *K. oboediens*. Thus, it is possible that functional genetic elements of *K. rhaeticus* were identified as gene contents of *K. oboediens* in short-read based analysis. Forty-one genetic elements were common among three *Komagataeibacter* MAGs. Three MAGs contained QS operons, which are essential for making the microbiome act like a multicellular single organism [28]. The QS system could play a role in the communication between microorganisms within KMC samples in harsh conditions, including UV radiation, high amount CO_2_, lack of water resource, and limitation of nutrient availability [29, 30]. Therefore, the QS system could be one of the driving factors for microbial survival in KMC samples.

Five unique genetic elements in *K. hansenii* could provide clue for tolerance to harsh environment at top-layer KMC sample. The type II TA system has been reported as an important system for stabilizing microbial community in stressed environment [31, 32]. Microorganisms in KMC sample could be diminished during long-term experiments by strong external stressors, and survival members had the advantage for growth, including space and nutrients supply in the limited environment by programmed cell death mediated by the TA system. The PPDK pathway is a CAM-light pathway that occurs in the cytosol with the proton gradient for ATP production occurring across the bacterial cytoplasmic membrane [33]. Microbiota in KMC samples can survive by using the PPDK pathway, which can be activated alternative carbohydrate fixation under high CO_2_ condition and dry environment with low oxygen concentration similar to Mars-like environment [34, 35]. The enzymes in the betaine biosynthesis pathway act in the biosynthesis of glycine betaine from choline. The glycine betaine is an efficient osmotic solute found in a wide range of bacteria, and this compound accumulates in response to osmotic stress as an osmoprotectant. Osmotic stress is one of the major stress factors in KMC outside the ISS as well as UV radiation, the glycine betaine is possible to protect KMCs against Mars-like environmental stress [36, 37]. In addition, glycine betaine could be a vigorous protectant against mutagenic sources such as radiation-induced damage [38]. The polyamines are organic alkaline compounds having multiple amino groups with a net positive charge at physiological pH. Because of their unique charge-structure, polyamines play a crucial role in the maintenance of negatively charged nucleic acids and are essential for normal cellular growth and multiplication [39]. Some polyamines, such as putrescine, modulate numerous cellular processes, including transcription and translation [40]. The predicted polyamine biosynthesis pathway in *K. hansenii* kmcMAG001 could produce putrescine by ornithine decarboxylase or arginine decarboxylase. Although further understanding of the mechanisms is necessary, the synthesis of polyamines could be an important factor for microbiome stabilization. These functional gene elements of *K. hansenii* MAG could be a clue for tolerance for harsh condition and protection other microbes in KMCs exposed to Mars-like conditions with a low level of water and UV radiating environments.

The unique genetic content of *K. oboediens* kmcMAG003 was the sulfate-sulfur assimilation pathway. Sulfur is an essential element in several metabolisms, including the amino acids methionine and cysteine, vitamin B7 (biotin), and glutathione [41, 42]. Therefore, the sulfate-sulfur assimilation pathway of *K. oboediens* could supply sulfur resource to microbes in KMCs. However, this unique pathway could not fully explain why *K. oboediens* had achieved more successful flourish in nutrient-rich and mild conditions than *K. hansenii* during re-adaptation. *K. oboediens* has eleven more functional elements than *K. hansenii*. This indicates that *K. oboediens* could be more functionally complex organism than *K. hansenii*. Competitive relationship between two species could not be applied in this case, because the relative abundance of *K. hansenii* was maintained and it was dominant in middle and bottom-layer samples. Therefore, *K. hansenii* in top-layer KMC sample was tolerated to harsh condition and could be reactivated immediately after return from ISS. However, strong stressors were influenced on *K. hansenii* at top-layer and this species could not flourish during readaptation.

In conclusion, the microbiome in KMCs exposed to Mars-like conditions was changed, but the dominant *Komagataeibacter* spp. especially *K. hansenii*, *K. rhaeticus*, and *K. oboediens* tolerated and could protect other microbes in KMC by retaining their genetic properties and active specific pathways. *K. oboediens* is the dominant member in KMC exposure to space condition during repeated cultivation. Our results can extend the understanding the microbiome in Kombucha exposed to extra-terrestrial conditions towards developing a KMC with minimal microbial composition.
